# Supplementary material for: A 3D-printed flow-cell for on-grid purification of electron microscopy samples directly from lysate
Source: J Struct Biol. Author manuscript; Available in PMC 2025 Dec 9. (PMC7618051; doi:10.1016/j.jsb.2023.107999)
Supplement: Supplementary Material [file EMS207957-suppement-Supplementary_Material.pdf]

Supplementary Materials

A 3D-printed flow-cell for on-grid purification of electron microscopy samples directly from lysate

Kailash Ramlaul[1], Ziyi Feng[1], Caoimhe Canavan[1], Natàlia de Martín Garrido[1], David Carreño[1], Michael Crone[1], Kirsten E. Jensen[1], Bing Li[2], Harry Barnet[3], David T. Riglar[1,4], Paul S. Freemont[1], David Miller[3†] & Christopher H. S. Aylett[1†]

[1] Section for Structural and Synthetic Biology, Department of Infectious Disease, Imperial College London, London, United Kingdom.

[2] Hamlyn Centre, Department of Brain Sciences, Imperial College London, London, United Kingdom.

[3] Imperial College Advanced Hackspace, Imperial College London, London, United Kingdom.

[4] The Francis Crick Institute, London, United Kingdom.

† To whom correspondence should be addressed:

C.H.S.A. c.aylett@imperial.ac.uk

D.M. d.miller@imperial.ac.uk

Supplementary Figure 1

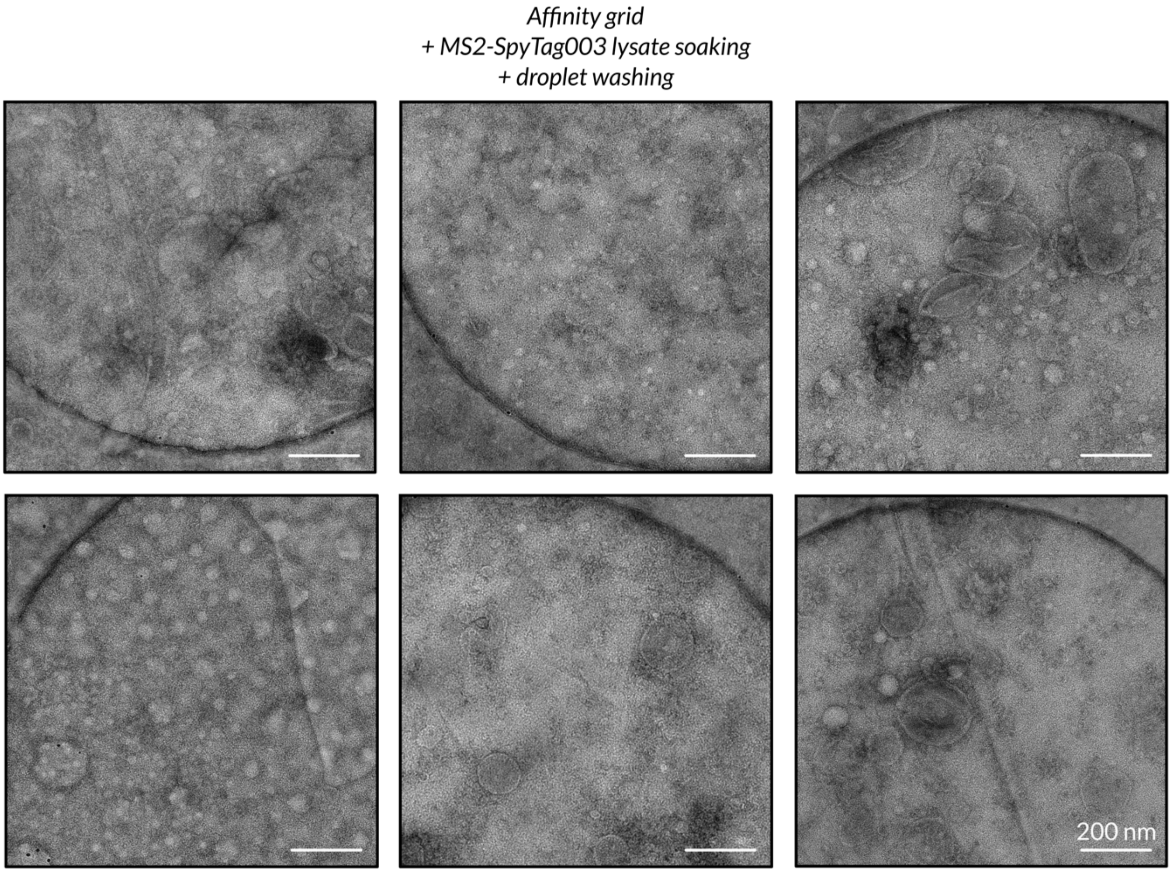

Supplementary Figure 1: Control experiment showing the results of attempted on-grid purification from lysate without the use of our flow-cell. Representative images from three independently prepared samples showing the results obtained upon incubation of our affinity grids for 1.5 hours with light agitation on ten-fold diluted cell-lysate from BL21(DE3) *E. coli* expression of our MS2-Spycatcher003 coat protein construct, followed by three-fold serial washing with droplets containing wash buffer, and finally negative staining with 2% (w/v) uranyl acetate. Extensive contamination and numerous aggregates are visible, as well as ruptured cell envelopes. Affinity captured MS2 capsids can be identified in some images, however density is low compared to the results we report in Figure 5, and these samples are unsuitable for further characterisation in vitreous ice or for single-particle analysis of the affinity-captured particles.
